# Identification of novel mutation in cathepsin C gene causing Papillon-Lefèvre Syndrome in Mexican patients

**DOI:** 10.1186/1471-2350-14-7

**Published:** 2013-01-11

**Authors:** José G Romero-Quintana, Luis O Frías-Castro, Eliakym Arámbula-Meraz, Maribel Aguilar-Medina, Jesús E Dueñas-Arias, Jesús D Melchor-Soto, José G Romero-Navarro, Rosalío Ramos-Payán

**Affiliations:** 1 Faculty of Biological and Chemical Sciences, Doctoral Program in Biotechnology and Master Program in Biomedical Sciences, Autonomous University of Sinaloa, Culiacán, Sinaloa, 80010, México; 2 Dermatology, Pediatric Hospital of Sinaloa, Culiacán, Sinaloa, 80200, México; 3 Genetic, Pediatric Hospital of Sinaloa, Culiacán, Sinaloa, 80200, México; 4 Odontopediatry divisions, Pediatric Hospital of Sinaloa, Culiacán, Sinaloa, 80200, México

**Keywords:** Papillon-Lefèvre Syndrome, Cathepsin C, Mutations, Enzymatic activity, HLA, Mexicans

## Abstract

**Background:**

Papillon-Lefèvre Syndrome (PLS) is a type IV genodermatosis caused by mutations in cathepsin C (CTSC), with a worldwide prevalence of 1–4 cases per million in the general population. In México, the prevalence of this syndrome is unknown, and there are few case reports. The diagnosis of twenty patients in the state of Sinaloa highlights the need to characterize this syndrome in Mexicans.

**Methods:**

To understand the basis of PLS in Mexicans, the gene expression, enzymatic activity and mutational analysis of CTSC were assayed in nine PLS patients and their relatives. Frequencies of *CTSC* gene polymorphisms and HLA alleles were determined in these patients, their relatives, and the population.

**Results:**

Patients showed normal *CTSC* gene expression, but a deep reduction (up to 85%) in enzymatic activity in comparison to unrelated healthy individuals. A novel loss-of-function mutation, c.203 T >; G (p.Leu68Arg), was found in all patients, and some carried the polymorphism c.458C >; T (p.Thr153Ile). Allelic frequencies in patients, relatives and controls were 88.89%, 38.24% and 0.25% for G (c.203 T >; G); and 11.11%, 8.82% and 9.00% for T (c.458C >; T). HLA-DRB1*11 was found significantly more frequent (*P* = 0.0071) in patients than controls (33.33% *vs.* 7.32%), with an estimated relative risk of 6.33.

**Conclusions:**

The novel loss-of function mutation of *CTSC* gene (c.203 T >; G) found in patients correlated with their diminished enzymatic activity, and HLA-DRB1*11 was found to be associated with PLS. The study of more PLS patients may give more insights into the etiology of the disease as well as its prevalence in México.

## Background

Papillon-Lefèvre Syndrome (PLS) is an autosomal recessive disorder that is caused by mutations in cathepsin C (CTSC) (OMIM #245000). PLS is characterized by palmoplantar hyperkeratosis and aggressive periodontitis, has a worldwide prevalence of 1–4 cases per million in the general population, and is often related with consanguinity [1-3]. Dermatological disorders initiate with erythema and after about six months, they progress to hyperkeratosis of soles, palms, knees and elbows [4]. This exacerbates in winter, leading to painful fissures. Aggressive periodontitis begins with the primary dentition, leading to periodontium destruction, and the premature loss of deciduous teeth by age of 6 years; afterwards gums heal, until the development of the permanent dentition, when periodontitis reappears and teeth are prematurely lost by the age of 16-years [5]. Besides these basic features, PLS patients can present with liver abscesses [6-9], increased incidence of pyogenic infections [10], or mental retardation [11]. Medication for hyperkeratosis and periodontal care improve quality of life [12,13].

The *CTSC* gene (11q14.2) spans 4.7 kb and has seven exons [14,15]. *CTSC* gene mutations have been reported as responsible for PLS [14], as well as similar conditions such as Haims-Munk Syndrome, and juvenile periodontitis [16,17]. CTSC is expressed at high levels in polymorphonuclear leukocytes, alveolar macrophages, skin, kidney, and placenta, and at moderate or low levels in a variety of other organs. Gene structure shows several possible tissue-specific regulatory elements.

CTSC (EC 3.4.14.1) or dipeptidyl peptidase I (DPPI) is a lysosomal cysteine protease that removes dipeptides from the free N-termini of protein and peptides. The *CTSC* gene encodes a polypeptide chain that folds into an exclusion domain, a propeptide, and two papain-like domains [18,19]. The active CTSC is a tetramer (200 kD) [20,21], while cathepsin B, H and L are monomers [21-25]. CTSC plays a major role in the cleavage of some hormones (including glucagon, gastrin and angiotensin II), and the activation of granule serine proteases from cytotoxic T lymphocytes, natural killer cells (granzymes A and B), mast cells (tryptase and chymase), and neutrophils (cathepsin G and elastase) [19,20,26-28].

CTSC deficiency has been associated with PLS-associated increased susceptibility to bacterial infection in gums and other sites; however, the heterogeneous severity of the periodontitis and the susceptibility to infections observed in PLS suggests the existence of compensatory pathways in most tissues [29]. The PLS phenotype also suggests a role for CTSC in epithelial differentiation or desquamation. Several works have shown a relationship between CTSC and the development of gingival fibroblasts, and the peeling of skin keratin in affected zones [30]. Aberrant epithelial differentiation may affect the junctional epithelium that binds the gingiva to the tooth surface, possibly weakening this mechanical barrier and allowing deeper colonization by periodontal pathogens [30].

The Major Histocompatibility Complex (MHC) *loci* (6p21.31) span nearly 3.6 Mb, and contains the most polymorphic human genes, with strong linkage disequilibrium. MHC products, known as Human Leukocyte Antigens (HLA) class I (A, B and C) and class II (DP, DQ and DR), play a key role in antigen presentation to T-cells during elicitation of adaptive immune responses [31]. Some PLS patients show an increased susceptibility to recurrent suppurative infections, and several works have attempted to associate HLA alleles with PLS and severe periodontitis [32-34].

In México, the prevalence of PLS is unknown. Sada-Tamayo *et al.* reported three cases of this syndrome in the Mexican state of Tamaulipas in 2007 [35]. We have found twenty PLS patients in the state of Sinaloa, indicating a high prevalence of the disease in this region. In this work, we report the clinical characteristics, gene expression, enzymatic activity and mutational analysis of CTSC in nine PLS patients (from seven different families) and their relatives. Frequencies of *CTSC* gene polymorphisms and HLA alleles were determined in these patients, their relatives and the population.

## Methods

### Study subjects

We have diagnosed twenty patients with Papillon-Lefèvre Syndrome at the Hospital Pediátrico de Sinaloa (HPS) level 2, but only nine were available to participate in this study. These nine patients were born to seven non-related families from the following locations at the northwestern Mexican state of Sinaloa: La Reforma (two families), Las Aguamitas, Higueras de Abuya, Sinaloa de Leyva, Navolato and Culiacán. Parents (n = 12) and siblings (n = 5) of the patients, and unrelated healthy individuals (n = 8) were included in the study as comparisons. Voluntary blood donors (n = 200) from the Instituto Mexicano del Seguro Social (IMSS) were included for population analysis. All subjects were natives of Sinaloa for two previous generations. The Ethical and Research Committee of HPS approved this study and subjects signed an informed consent.

### Cell isolation

Peripheral blood mononuclear cells (PBMCs) and Polymorphonuclear cells (PMNs) were isolated by centrifugation on Polymorphprep (Axis Shield, Oslo, Norway) following manufacturer recommendations. Contaminating erythrocytes in pellets were lysed by adding 5 mL lipopolysaccharide (LPS)-free cold water for 45 seconds and immediately adding 5 mL of LPS-free cold physiological saline solution. Purity of cell fractions was assessed by directly counting Giemsa-stained smears. Cell viability was assessed by trypan blue exclusion-stain (Invitrogen, Carlsbad, CA, USA), and was always >;90% in all experiments of this work.

### Enzymatic activity

Leukocyte pellets were disrupted in 100 μL lysis buffer (10 mM Na_3_PO_4_, 0.1% Trixon X-100) by one pulse of sonication (100 W potency) for 5 seconds in ice, using an ultrasonic probe Sonic Dismembrator 100 (Fisher Scientific, Pittsburgh, PA, USA). Samples were centrifuged at 2,500 g during 5 min at 4°C, and the supernatant were recovered for enzymatic assays. Proteins were quantified using the Bradford assay.

Enzymatic activity was assayed by mixing 20 μL of cell extract with 30 μL reaction buffer (100 mM Na_3_PO_4_, 2 mM NaCl, 2 mM DTT), 30 μL dilution buffer (10 mM Na_3_PO_4_, 0.1% Trixon X-100), and 100 μL of chromogenic CTSC-substrate (1.5 mM gly-phe-para-nitroanilide). Absorbencies were measured at 405 nm, samples were incubated at 37°C for 1 h, and read again. Dipeptidyl peptidase activity of samples, expressed as para-nitroanilide released (μmol/mg of protein * hour), was determined interpolating the difference between first and second reads in a standard curve (0.025 to 175 μmol) of pure para-nitroanilide (pNA) (Sigma-Aldrich, St. Louis, MO, USA). Dipeptidyl peptidase activity from healthy individuals was set up as 100% and used for comparison to all other groups.

Bovine spleen cathepsin C (0.5 μg/μL solution with 5 μg/μL BSA) (Sigma-Aldrich) was used to determine enzyme kinetics by measuring pNA release from the substrate. A reaction rate of 2.58 ±0.47 μmol of pNA released per minute was estimated for pure bovine spleen CTSC, and was similar to samples rates of 1.66 ±0.22. Reaction conditions and inhibitor (iodoacetic acid, Sigma-Aldrich) concentrations were determined from kinetic assays and others reports [36,37].

### Quantitative analysis of gene expression by qPCR

Purified leukocyte pellets were homogenized in 1 mL of TRIZOL reagent (Invitrogen). Total RNA was extracted in accordance to manufacturer’s instructions, treated with DNase I (Invitrogen), and its integrity and concentration were confirmed by electrophoresis and spectrophotometry, respectively. RNA (2 μg) was annealed to Oligo-dT, and synthesis of single-strand complementary DNA (cDNA) was performed under standard conditions.

Quantitative Polymerase Chain Reaction (qPCR) was performed following conditions previously described [38], using a MiniOpticon Real-Time PCR Detection System (Bio-Rad, Hercules, CA, USA). Primers to amplify 273 bp (from exons 7 and 8) of human glyceraldehyde 3-phosphate dehydrogenase (GAPDH) gene (5’-TCATCCAT GACAACTTTGGTATCG-3’;   5’-TGGCAGGTTTTTCTAGACGGC-3’) and 125 bp (from exons 6 and 7) of *CTSC* gene (5’-AGGAGGTTGTGTCTTGTAGCC-3’;  5’-AGTGCCTGTGTAGGGGAAGC-3’) were designed with the Primer Express 2.0 software (Applied Biosystems, Foster City, CA, USA). Relative expression was calculated with the 2(−Delta Delta C(T)) method [39]. Expression level from healthy individual was set up as 1.0 for all comparisons.

### PCR-SSCP and sequencing

Genomic DNA was isolated from whole blood containing EDTA, using the salt precipitation method [40]. The seven exons of the *CTSC* gene, and their splice junctions, were amplified by PCR, under standard conditions using a PXE 0.2 thermocycler (Thermo Electron). Sequence of primers for exons 2 to 6 were taken from a previous report [14], and were designed for exons 1 (5’-CGCCTCGTGGTGGACTCAC-3’; 5’-GCTCAAGGG CAGAAAGGACG-3’) and 7 (5’-GCAAAGAATAATG GAGCAAAGAAGA-3’;  5’-AATTCCCCTTTACAACT GATGCA-3’).

Mutation scanning of *CTSC* exons was done by single-strand conformation polymorphism (SSCP), as described elsewhere [41,42]. Briefly, PCR product samples were diluted, 1:1 in loading buffer (95% formamide, 20 mM EDTA, 0.05% bromophenol blue, and 0.05% xylene cyanol), heated at 95°C for 5 min and immediately placed on ice. Aliquots were subjected to electrophoresis on polyacrylamide gel at 300 V for 3.2 h, at 10°C (±0.5). Gels were silver stained to visualize the reannealed single-stranded PCR products.

Amplified exons with mobility shifts were analyzed to identify nucleotide changes by automated DNA sequencing. Obtained sequences were compared with human CTSC sequence reference of GenBank (NG_007952.1) using CLC sequence viewer V6.4 software (CLC bio, Aarhus, Denmark).

### Protein homology analysis

Amino acid sequences of CTSC from human (NP_001805), rhesus monkey (XP_001104734), cattle (AAI02116), dog (AAD02704), house mouse (AAH67063), Norway rat (NP_058793), zebrafish (AAH64286), African clawed frog (NP_001080511), black tiger shrimp (ABW74905), Pacific white shrimp (ACK57788), *Marsupenaeus japonicum* (BAC57943) and *Schistosoma japonicum* (ACC32040), were obtained from GenBank. Sequences were aligned and analyzed using CLC sequence viewer (CLC bio).

Amino acid substitutions were analyzed with the PolyPhen-2 (Polymorphism Phenotyping v2) software (http://genetics.bwh.harvard.edu/pph2) to predict their possible functional effect, in a scale from 0.00 (for benign) to 1.00 (for deleterious).

### PCR-RFLP

Exons 2 and 3 were amplified by PCR followed by restriction fragment length polymorphism (RFLP). A region of 339 bp from exon 2 of *CTSC* gene, containing the c.203 T >; G mutation, was amplified with the same primers used for SSCP, and digested with *Acu I* (1 U for 2 h at 37°C), to identify wild-type T allele (202 and 139 bp fragments) and the mutated G (loss of enzyme recognition site). In the same way, a region of 257 bp from exon 3, spanning the polymorphic site c.458C >; T, was amplified using a modified reverse-primer (5’-TCCTGAGAATTCTTAAGGTCAGC-3’) to generate a recognition site for *Pvu II*. After digestion (1 U for 2 h at 37°C), normal C allele generates a 235 bp and a 22 bp fragments, while allele T remains uncleaved. PCR products and digestion fragments were visualized in silver-stained polyacrylamide or GelRed-stained (Biotium, Hayward, CA, USA) agarose gels.

### HLA typing

Molecular typing of HLA class I (HLA-A, HLA-B) and HLA class II (HLA-DRB1, HLA-DQB1), at low resolution procedure modality, was done by PCR sequence-specific primers (PCR-SSP) technique, with the AB/DR/DQ SSP UniTray kit, (Dynal, Invitrogen) following manufacturer’s instructions, and using GoTaq Flexi DNA Polymerase (Promega, Madison, WI, USA). Products were separated by electrophoresis in 2% agarose. UniMatch Plus 5.0 software (Invitrogen) was used to type the specific alleles.

### Statistical analysis

Experimental data was obtained from triplicates and presented as mean ± SD. Comparisons in gene expression and enzymatic activity were performed with ANOVA and Bonferroni multiple comparison test, and *P* values of less than 0.05 were considered statistically significant. A population sample of 96 individuals was calculated to estimate the frequency of *CTSC* gene polymorphisms in the population. Differences in allele frequencies were evaluated by χ^2^ test or Fisher’s exact test. Significant *P* values (≤ 0.05) were corrected taking into account the number of alleles observed [43]. Odds ratios (OR) with 95% confidence intervals (CI) were used as the measure of association between specific alleles with PLS [44]. Hardy-Weinberg’s equilibrium was calculated by χ^2^ test. PASW v18.0 (SPSS inc., Chicago, IL, USA) and Arlequin v3.5.1.2 (Swiss National Science Foundation) software packages were used for analysis.

## Results

### Clinical evaluation

The nine PLS-diagnosed patients described in this work showed keratosis palmoplantaris, with affected knees and elbows, and aggressive periodontitis with variable presentation. They were born (first or second gestation) to non-consanguineous parents. Ages ranged from 1.5 to 61 years (median = 9), 55.56% were female and 44.44% male. Panoramic and periapical radiographs confirmed the alveolar bone loss in periodontal disease, and were used to follow-up with patients after dental treatments. Some patients showed increased susceptibility to pyoderma, such as furuncles and cold abscesses. Standard microbiologic tests showed the presence of *Aggregatibacter actinomycetemcomitans* and *Staphylococcus aureus* in lesions. Infections were treated with clindamycin with positive results. Periodontitis and hyperkeratosis severities did not correlate. Hyperkeratosis was treated with topical keratolytics containing salicylic acid and urea, resulting in notable improvement of condition. Routine blood and biochemical laboratory tests were within normal limits.

**Figure 1:**
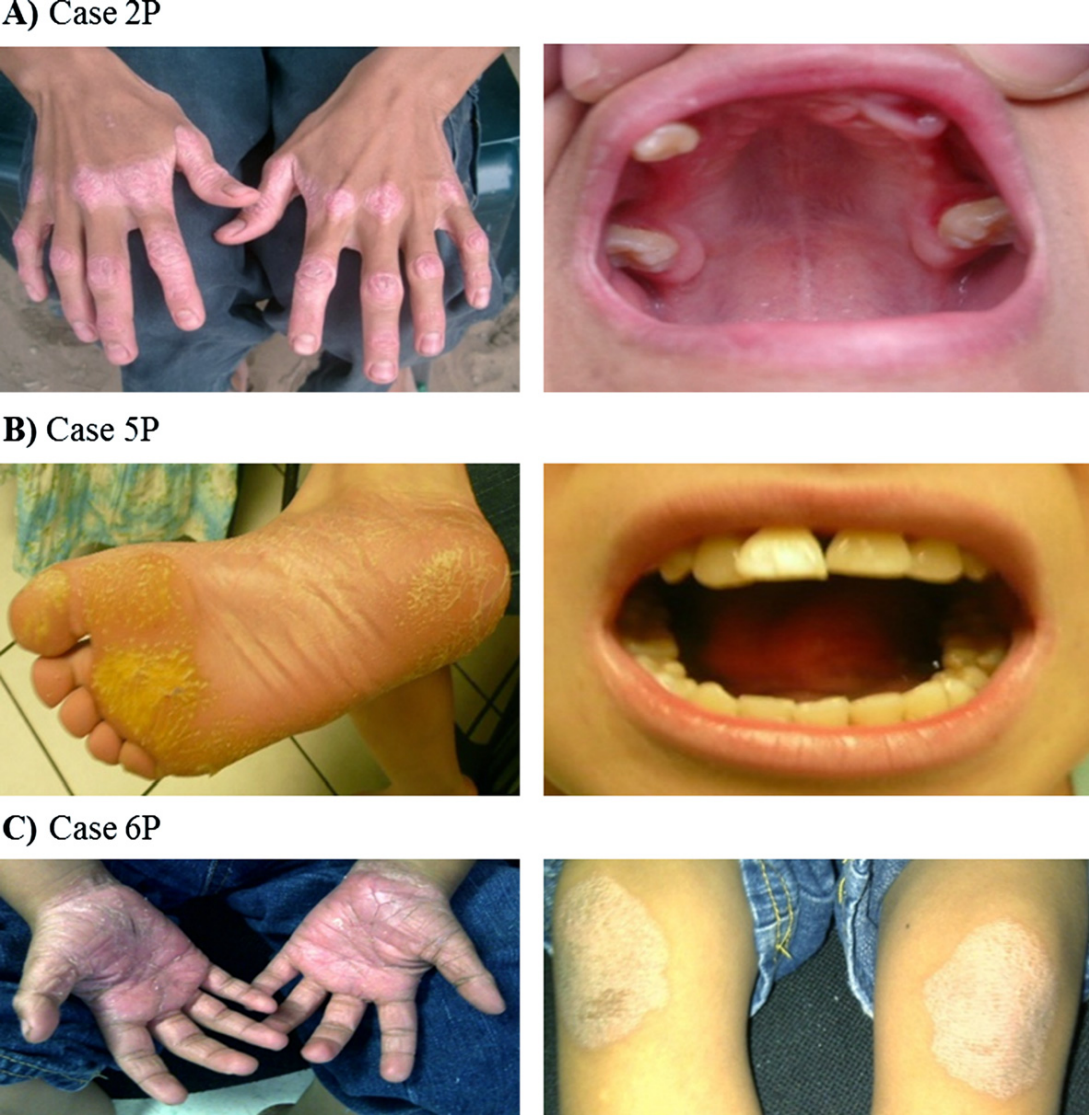
**Representative clinical features of patients with Papillon-Lefèvre Syndrome. A**) Case 2P: Overt hyperkeratosis in hands (dorsal view). With gingivitis, missing teeth and periodontal pocket. **B**) Case 5P: Mild hyperkeratosis of sole and mobile teeth. **C**) Case 6P: Psoriasiform lesions over palms and knees.

*Case 1P1:* 7 year-old female, showed overt palmoplantar hyperkeratosis, with fissures. She had only one deciduous tooth remaining, but her gums still showed signs of swelling and inflammation. She suffered recurrent folliculitis and furuncles. *Case 1P2:* 11 year-old female, with mild hyperkeratosis. She had severe periodontitis, and suffered recurrent bacterial skin infections. *Case 1P3:* 61 year-old male (uncle of the father of cases 1P1 and 1P2), with mild hyperkeratosis. He had lost all his permanent teeth, and gums appeared healthy. *Case 2P:* 14 year-old male (Figure 1A), showed overt hyperkeratosis and severe periodontitis. X-ray films of both hands discarded arachnodactyly, discarding Haims-Munk Syndrome. *Case 3P:* 6 year-old female, with mild hyperkeratosis. She developed periodontitis just after initiation of primary dentition. *Case 4P:* 12 year-old female, showed mild hyperkeratosis and severe periodontitis, and lost most of the permanent teeth. *Case 5P:* 12 year-old female (Figure 1B), with mild hyperkeratosis. She had periodontitis and loss of many permanent teeth, malformed and decaying teeth were observed. *Case 6P:* 3 year-old male (Figure 1C), showed overt hyperkeratosis. Primary dentition has not occurred yet and gums appeared healthy. *Case 7P:* 18-month-old male, first gestation, showed mild hyperkeratosis. Primary dentition has not occurred yet and gums appeared healthy.

### CTSC activity and gene expression

Enzymatic activity and gene expression (Table 1) were measured in PLS patients (n = 7), their relatives (n = 17) and unrelated healthy individuals (n = 8). Patients showed 14.89% (±4.96) of the enzymatic activity observed by controls (100%), while parents and siblings showed 47.60% (±19.74) and 38.42% (±24.90), respectively. This represents an enzymatic deficiency of nearly 85% in patients, and nearly 50% in parents and siblings.

**Table 1:** Percentage of cathepsin C activity, gene expression, and genotypes in patients with Papillon-Lefèvre Syndrome and their relatives

**Family**	**Subject**	**%**	**SD**	***P***	**CE**	**SD**	***P***	**c.203 T >; G**	**c.458C >; T**
**1**	1P1	13.45	2.97	0.0001	0.25	0.05	0.0070	G/G	C/C
	1P2	-	-	-	-	-	-	G/G	C/C
	1P3	-	-	-	-	-	-	G/G	C/C
	1 F	46.99	1.40	0.0041	1.62	0.05	0.0110	T/G	C/C
	1 M	67.93	2.76	0.0489	0.31	0.11	0.0170	T/G	C/C
**2**	2P	11.03	4.41	0.0061	1.81	0.09	0.0750	T/G	C/T
	2 F	59.22	5.76	0.1580	2.99	1.07	0.1190	T/G	C/C
	2 M	42.91	5.01	0.0561	0.42	0.09	0.1190	T/T	C/T
	2B1	39.26	3.25	0.0438	0.37	0.06	0.1000	T/T	C/T
	2B2	-	-	-	1.33	0.01	0.1440	T/T	C/C
**3**	3P	24.16	1.33	0.0000	1.39	0.66	0.4280	G/G	C/C
	3 M	55.76	0.90	0.0000	0.44	0.07	0.0070	T/G	C/C
**4**	4P	14.97	0.10	0.0013	0.19	0.01	0.0080	T/G	C/T
	4 M	27.75	0.10	0.0018	1.40	0.08	0.0470	T/T	C/T
	4S	72.64	0.16	0.0106	0.57	0.11	0.0560	T/G	C/C
	4B	26.93	0.17	0.0017	0.29	0.11	0.0210	T/G	C/C
**5**	5P	15.32	2.12	0.0000	0.15	0.00	0.0570	G/G	C/C
	5 F	77.74	12.07	0.0608	2.55	1.81	0.2900	T/G	C/C
	5 M	56.50	1.26	0.0016	0.46	0.05	0.1260	T/G	C/C
**6**	6P	10.42	0.86	0.0000	0.34	0.12	0.1030	G/G	C/C
	6 F	16.92	2.01	0.0000	1.37	0.57	0.0177	T/G	C/C
	6 M	24.23	7.85	0.0000	1.32	0.39	0.0123	T/G	C/C
	6S	14.84	3.61	0.0000	0.34	0.21	0.0002	T/G	C/C
**7**	7P	-	-	-	2.15	0.10	0.0410	G/G	C/C
	7 F	-	-	-	0.57	0.18	0.2220	T/G	C/C
	7 M	-	-	-	1.06	0.79	0.8020	T/G	C/C

%, percentage of enzymatic activity in relation to healthy individuals (set as 100%). CE, cathepsin C expression in relation to healthy individuals (set up as 1.0). *P*, p value from comparison to healthy individuals. (−), sample not available. Subjects: P, patient; F, father; M, mother; B, brother: S, sister. SD, standard deviation.

Quantification of *CTSC* expression showed no significant differences (*P* >; 0.05) among all groups. Relative expression in comparison to controls (set up as 1.0) was 0.90 (±0.86), 1.21 (±0.86) and 0.58 ±0.43 for patients, parents and siblings, respectively.

### Mutational analyzes of CTSC

**Figure 2:**
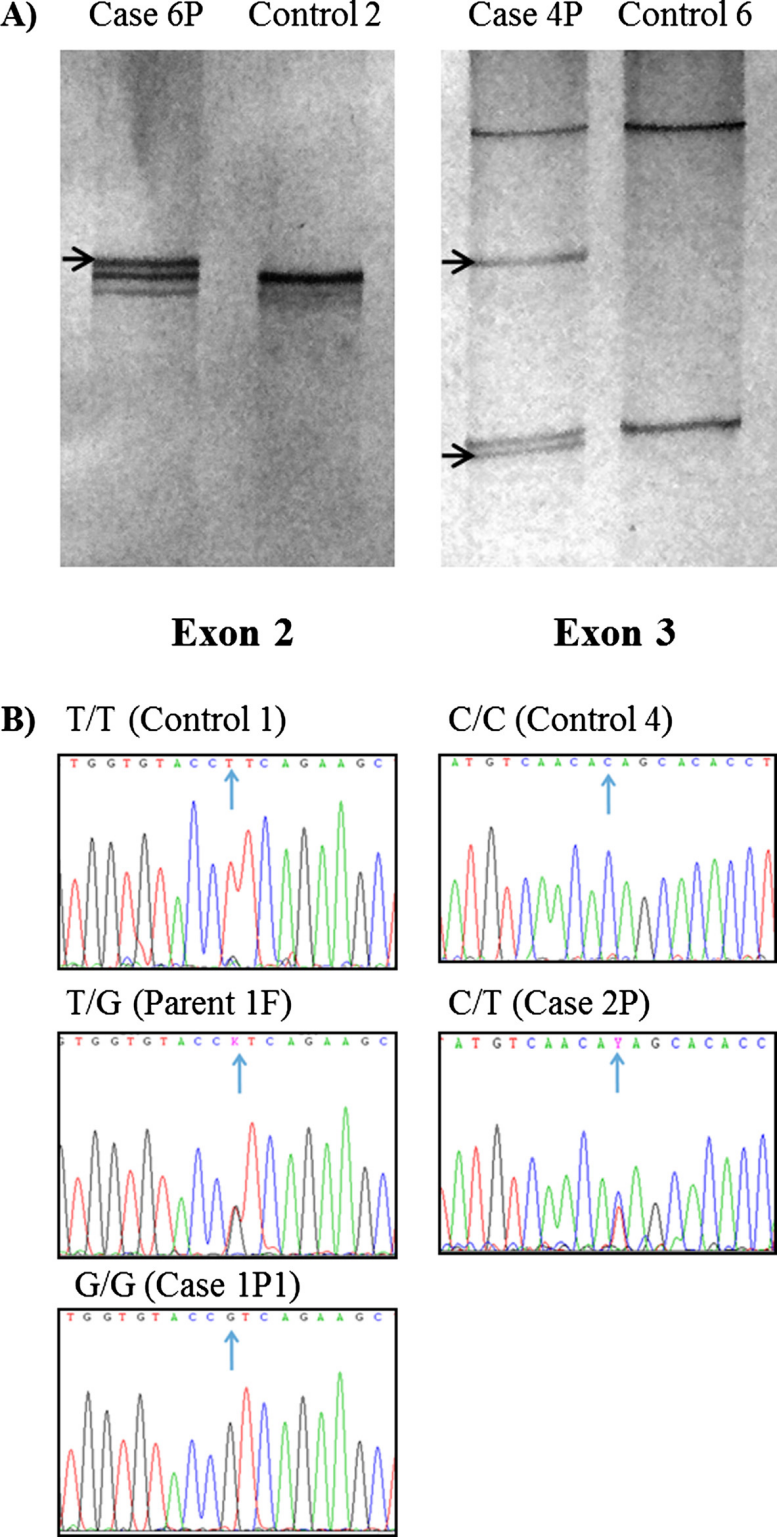
**Mutational analysis of Cathepsin C gene. A)** Representative results of *CTSC* gene exon scanning by single-strand conformation polymorphism (SSCP). Reannealed products, in silver-stained polyacrylamide gels, of exons 2 (left) and 3 (right) in cases 6P and 4P showed differences (arrows) against controls 2 and 6. **B)** Representative electropherograms of exon 2 showing genotypes (arrows) homozygote normal T/T (control 1), heterozygote T/G (parent 1 F) and homozygote mutated G/G (case 1P1), for mutation c.203 T >; G. Exon 3, showing genotypes (arrows) homozygote normal C/C (control 4) and heterozygote C/T (case 2P) for polymorphism c.458C >; T.

The coding sequences and splice junctions of *CTSC* gene were analyzed by SSCP in nine PLS patients and their relatives. There were changes in reannealed products in exons 2 and 3 from patients and relatives (Figure 2A). As expected, there were no changes in any exon in eight unrelated healthy individuals. On sequencing (nt 177 to 228 of exon 2, and nt 433 to 484 of exon 3), nucleotide changes were found at positions 203 (T >; G) of exon 2 and 458 (C >; T) of exon 3 (Figure 2B). All sequence variants were submitted to GenBank nucleotide database (JQ763324-77) and to dbSNPs (rs199474831).

Cases 2P and 4P were found to be compound heterozygous for mutations c.203 T >; G and c.458C >; T, while all other patients were found to be homozygous (G/G) for mutation c.203 T >; G. All parents and four siblings were carriers of either c.203 T >; G or c.458C >; T, but not affected (Table 1).

### Protein homology analysis

DNA sequence analysis revealed that c.203 T >; G mutation is located in the exclusion domain of DPPI, resulting in a change of the amino acid Leucine for Arginine at residue 68 (p.Leu68Arg). The presence of Arginine at this site may interfere with the insertion of the dipeptide substrate, subsequently blocking the enzymatic activity. In fact, homology analysis showed that Leucine 68 is a highly conserved residue, and analysis of this substitution with the PolyPhen-2 software predicted a damaging mutation, with a score of 1.00.

The substitution c.458C >; T results in a change of the amino acid Threonine for Isoleucine at residue 153 (p.Thr153Ile); this residue is not highly conserved in evolution and is located in the pro-region of heavy chain, which is removed during DPPI activation. PolyPhen-2 software predicted a probably benign mutation with a score of 0.00.

### Population frequencies of *CTSC* gene polymorphisms

**Figure 3:**
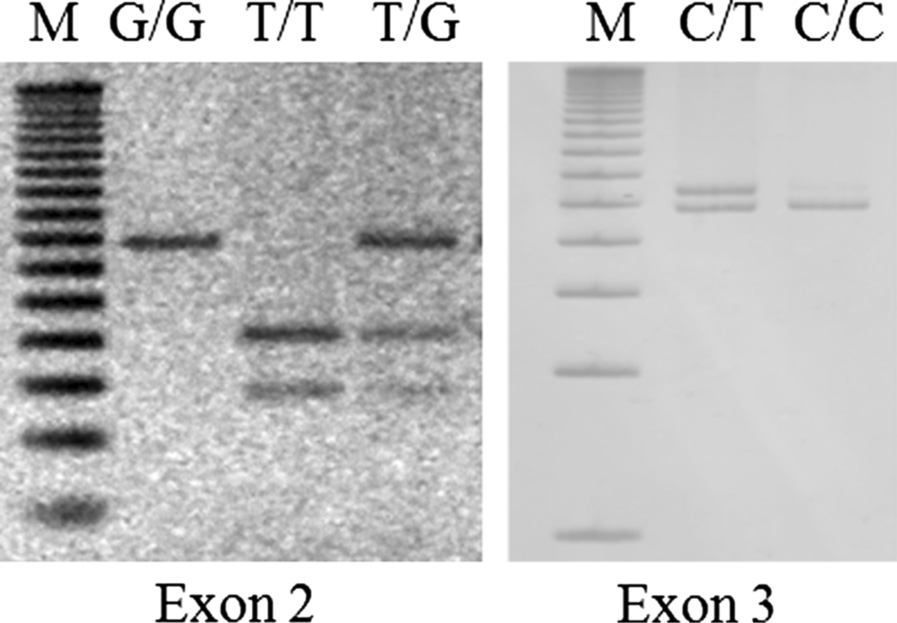
**Gene polymorphisms analysis.** PCR-RFLPs products of genotypes homozygous normal T/T, heterozygous T/G and homozygous mutated G/G for mutation c.203 T >; G of exon 2 (left). Genotypes homozygous normal C/C and heterozygous C/T, for c.458C >; T polymorphism of exon 3 (right). M, 50 bp DNA ladder (Invitrogen).

PCR-SSCP analyzes of *CTSC* exons 2 and 3 were done in individuals from the population (n = 200). Positive samples were confirmed for mutations c.203 T >; G and c.458C >; T by PCR-RFLPs (Figure 3).

Allelic frequencies in PLS patients (n = 9), their relatives (n = 17) and controls were 88.89%, 38.24% and 0.25% for G (c.203 T >; G), respectively; and 11.11%, 8.82% and 9.00% for T (c.458C >; T). Genotypes frequencies of c.203 T >; G were 77.78%, 0.0% and 0.0% for G/G; 22.22%, 76.47% and 0.5% for T/G; and 0.0%, 23.53% and 99.5% for T/T. For c.458C >; T genotypes, frequencies were 22.22%, 17.65% and 18.0% for C/T; and 77.78%, 82.35, and 82.0% for C/C (Table 2). The distributions of c.203 T >; G and c.458C >; T polymorphisms in the groups were in Hardy-Weinberg equilibrium (all *P* >; 0.05).

**Table 2:** Allelic (af) and genotypic (gf) frequencies of cathepsin C polymorphisms in patients with Papillon-Lefèvre Syndrome, their relatives and controls

**Polymorphism**	**Patients**		**Relatives**		**Controls**	
**c.203 T >; G**						
Alleles	n = 18	af	n = 34	af	n = 400	af
G	16	0.8889	13	0.3824	1	0.0025
T	2	0.1111	21	0.6176	399	0.9975
Genotypes	n = 9	gf	n = 17	gf	n = 200	gf
G/G	7	0.7778	0	0.0000	0	0.0000
T/G	2	0.2222	13	0.7647	1	0.0050
T/T	0	0.0000	4	0.2353	199	0.9950
**c.458C >; T**						
Alleles	n = 18	af	n = 34	af	n = 400	af
T	2	0.1111	3	0.0882	36	0.0900
C	16	0.8889	31	0.9118	364	0.9100
Genotypes	n = 9	gf	n = 17	gf	n = 200	gf
T/T	0	0.0000	0	0.0000	0	0.0000
C/T	2	0.2222	3	0.1765	36	0.1800
C/C	7	0.7778	14	0.8235	164	0.8200

For c.203 T >; G there were significant differences in allelic (*P* = 0.0000; OR = 3192.00; 95% CI = 274.92-37061.06) and genotypic (*P* = 0.0000) frequencies between patients and population controls and in alleles (*P* = 0.0000; OR = 247.00; 95% CI = 30.83-1978.58) and genotypes (*P* = 0.0000) between patients’ relatives and controls. For c.458C >; T alleles and genotype distributions there were no significant differences between all groups (all *P* >; 0.05).

The initial calculated sample size for population analysis was 96, however, an individual carrying a heterozygous T/G genotype for c.203 T >; G was found, and therefore a final sample of 200 individuals was used.

### Immunogenetics

HLA alleles were typed in PLS patients (n = 9), their relatives (n = 15) and population controls (n = 41) (Table 3). The more common HLA alleles found in patients and controls were 33.33% *vs.* 14.63% (*P* = 0.0873; OR = 2.92; 95% CI = 0.92-9.26) for HLA-A*24, and 16.67% *vs.* 29.27% (*P* = 0.2755) for A*02. HLA-B*35 was found in 33.33% *vs.* 20.73% (*P* = 0.2500), and B*44 was found in 11.11% *vs.* 10.98% (*P* = 0.9867). Frequencies of HLA-DRB1*11 were 33.33% *vs.* 7.32% (*P* = 0.0071; OR = 6.33; 95% CI = 1.75-22.89), and 22.22% *vs.* 24.39% (*P* = 0.8454) for DRB1*04. Frequencies for HLA-DR53, DR52 and DR51 were 44.44% *vs.* 41.46% (*P* = 0.8165), 33.33% *vs*. 21.95% (*P* = 0.3059), and 22.22% *vs*. 24.39% (*P* = 0.8454), respectively. For HLA-DQB1*0301, frequencies were 33.33% *vs.* 24.39% (*P* = 0.2715), and 27.08% *vs.* 17.07% (*P* = 0.1745) for DQB1*0302. Significant deviations from Hardy–Weinberg equilibrium were not detected in the distribution of HLA alleles (all *P* >; 0.05).

**Table 3:** Allele frequencies (af) of the more common HLA class I and II molecules found in patients with Papillon-Lefèvre Syndrome, their relatives and controls

**Alleles**	**Patients**		**Relatives**		**Controls**	
	**(N = 18)**		**(N = 30)**		**(N = 82)**	
**HLA-A**		**af**		**af**		**af**
A*24	6	0.3333	8	0.2667	12	0.1463
A*02	3	0.1667	4	0.1333	24	0.2927
A*33	3	0.1667	2	0.0667	1	0.0122
A*31	2	0.1111	5	0.1667	8	0.0976
A*03	2	0.1111	2	0.0667	2	0.0244
**HLA-B**						
B*35	6	0.3333	9	0.3000	17	0.2073
B*65	3	0.1667	2	0.0667	2	0.0244
B*44	2	0.1111	2	0.0667	9	0.1098
B*51	1	0.0556	2	0.0667	4	0.0488
B*60	1	0.0556	2	0.0667	3	0.0366
**HLA-DRB1**						
DRB1*11	6	0.3333	6	0.2000	6	0.0732
DRB1*04	4	0.2222	9	0.3000	20	0.2439
DRB1*01	3	0.1667	6	0.2000	13	0.1585
DRB1*15	3	0.1667	4	0.1333	3	0.0366
DRB1*07	1	0.0556	2	0.0667	8	0.0976
**HLA-DQB1**						
DQB1*0301	7	0.3889	9	0.3000	20	0.2439
DQB1*0302	4	0.2222	9	0.3000	14	0.1707
DQB1*0501	4	0.2222	4	0.1333	9	0.1098
DQB1*0601	2	0.1111	2	0.0667	8	0.0976
DQB1*0201	1	0.0556	5	0.1667	14	0.1707

## Discussion

Here we described the clinical profile of nine patients (belong to seven different families) with Papillon-Lefèvre Syndrome (PLS), from the northwestern Mexican state of Sinaloa. They showed keratosis palmoplantaris and aggressive periodontitis with variable expression, which are the classical features of this syndrome [45]. In contrast to the nearly 30% of consanguinity of PLS cases reported in different countries [2,3], our PLS patients were born to non-consanguineous parents, and their families were not related.

PLS patients showed a DPPI activity reduction up to 85%, while parents and siblings showed a partial reduction of nearly 50%. This enzymatic deficiency was not due to diminished *CTSC* gene expression levels, as no significant differences were found between all groups. In agreement with our findings, more than 80% of all PLS cases reported show CTSC deficiency due to gene mutations [14,34]. Zhang *et al*. found less than 10% of the normal enzymatic activity in PLS patients and 50% in parents [46]. To our knowledge, there are no reports of CTSC messenger quantification using qPCR technique for this purpose in the literature.

Cases 2P and 4P were found to be compound heterozygous for c.203 T >; G and c.458C >; T, while all other cases were found to be homozygous for c.203 T >; G. Of note, all parents, as well as four siblings, carried either c.203 T >; G or c.458C >; T. These findings could explain the reduction in DPPI activity observed in these subjects, and seems to confirm the autosomal recessive mode of inheritance of PLS.

According to the Human Gene Mutation Database (HGMD), 77 variants of the *CTSC* gene have been reported. The c.203 T >; G substitution we report here, is a novel loss-of function mutation, and associates with the diminished enzymatic activity observed in our PLS patients. In accordance, frequency of allele G (c.203 T >; G) was higher in patients and relatives in comparison to population controls (all *P* ≤ 0.05).

The substitution c.458C >; T has been reported as both a common polymorphism in some populations, and as causal mutation in PLS patients [47-49]. c.458C >; T has been reported as causal mutation in one Australian PLS patient (compound heterozygous for c.458C >; T and c.199_222del) [49]. However, a genotypic study for the c.458C >; T polymorphism in the Spanish population reported frequencies of 81% for homozygote normal and 19% for heterozygote [47]. In accordance, allele T (c.458C >; T) was found relatively frequent in our population (9%), with no significant differences between the studied groups, and PolyPhen-2 software predicted a probably benign mutation. Therefore, the results of cases 2P and 4P remain inconclusive as the pathogenic variant other than c.203 T >; G remains unidentified in the coding region.

Some patients in this study showed increased susceptibility to bacterial skin infections. This condition seems to be due to an impairment of the immune system, which is involved in the pathoetiology of PLS. Therefore, we genotyped HLA class I and II alleles of PLS patients, their relatives and controls to evaluate any particular HLA association with this disease. We found fourteen HLA-A, twenty-nine HLA-B, twelve HLA-DRB1 and six HLA-DQB1 alleles (online Additional file 1), with HLA-A*02, B*35, DRB1*04, and DQB1*0301 being the most frequent in the population. A common HLA-A/B/DR/DP haplotype was not found in the families, confirming the lack of relationship among them.

With exception of HLA-DRB1*11, our results are in agreement with other reports showing no particular association of HLA alleles with PLS [32,34]. We found that HLA-DRB1*11 allele was significantly more frequent (*P* = 0.0071) in our PLS patients than in controls (33.33% *vs.* 7.32%, respectively), resulting in an estimated relative risk index of 6.33. Interestingly, this allele has been also associated with scleroderma in Caucasian patients [50]. HLA-A*24(9), HLA-B*35, DRB1*04 and DR51 showed higher frequency in our PLS patients than in controls, but the differences were not statistically significant. HLA-A*24(9) DRB1*04 and DR51 alleles have been associated with different kinds of periodontitis in other countries [33,51-53].

Mexicans, Mestizos in general, have a high degree of genetic heterogeneity due to centuries of mixing with Native Americans, Africans and Europeans. In the South and Central parts of Mexico, the predominant HLA type is more Amerindian than European or African, but in Sinaloa, half of the most common HLA haplotypes are believed to be of European origin. Allelic frequencies of the population found in this work were in accordance with a previous report [54]. An important difference is that our more extensive molecular study had included, for the first time, the side-by-side comparison of PLS families with unrelated, healthy controls.

Although the exact pathogenesis of PLS is still unknown, biochemical and genetic analysis allows for genetic counseling and opportune treatment, especially for those patients with familial history of this disease. Furthermore, some authors believe that if treatment is started at an early age, patients would possibly have normal adult dentition [55]. One of the studied cases (7P) is only 18-months-old, and will be under treatment and observation to avoid serious complications.

While in México the general prevalence of this syndrome is still unknown, and the number of reported cases is very low, the finding so far of 20 patients in the state of Sinaloa highlights the need for more studies of this nature. Due to the concentrated number of PLS patients found in our population, none of them previously described, is would be possible to argue that there is a founder effect for *CTSC* gene mutation. An haplotype analysis of DNA markers in *CTSC* gene region can help to determine if c.203 T >; G mutation has this founder effect.

## Conclusion

In conclusion, our work describes the immunogenetics, and the biochemical and molecular characterization of CTSC in nine PLS patients and their families. We describe the identification of a novel loss-of function mutation in *CTSC* gene (c.203 T >; G) causing PLS in Mexican patients; and the association of the HLA-DRB1*11 allele with this syndrome. The study of more PLS patients may give more insights into the etiology of the disease as well as its prevalence in México.

### Consent

Written consent was obtained from pateints’ parents for publication of this work.

## Competing interests

Authors declare that they have no competing interests.

## Authors’ contributions

LOFC, JEDA and JDMS diagnosed and treated the patients, and gave genetic counseling to the parents. JGRQ and JGRN conducted laboratory and biochemical test and interpreted results. EAM and MAM collected the data relative to this report. RPR and JEDA wrote the manuscript. All authors read and approved the final manuscript.

## Pre-publication history

The pre-publication history for this paper can be accessed here:

http://www.biomedcentral.com/1471-2350/14/7/prepub

## Supplementary Material

Additional file 1: Table S4 Complete HLA list. Allele frequencies (af) of HLA class I and II molecules, in studied subjects from Sinaloa, México. Click here for file
